# A long‐term comparison of radiographic and clinical outcomes between 62% and 70% weight‐bearing line targets in medial opening wedge high tibial osteotomy

**DOI:** 10.1002/jeo2.70900

**Published:** 2026-08-31

**Authors:** Woon Hwa Jung, Manan Paneliya, Saksham Goyal, Dong Hyun Kim, Ryohei Takeuchi

**Affiliations:** ^1^ Department of Orthopaedic Surgery Murup Hospital Changwon‐si Republic of Korea; ^2^ Department of Joint Surgery Yokohama Sekishinkai Hospital Yokohama Japan

**Keywords:** correction target, high tibial osteotomy, long‐term outcome, osteoarthritis, total knee arthroplasty, weight‐bearing line

## Abstract

**Purpose:**

The ideal intraoperative correction target for medial opening wedge high tibial osteotomy (MOWHTO) remains debated. This study compared 10‐year outcomes between two correction targets, 70% and 62% weight‐bearing line (WBL).

**Methods:**

A total of 103 knees that underwent MOWHTO with a locking plate, performed by one surgeon and randomized to a 70% (*n* = 50) or 62% (*n* = 53) WBL target, were retrospectively reviewed. Hip–knee–ankle angle and WBL were measured at fixed time points; clinical scores were recorded at 10 years. Conversion to total knee arthroplasty (TKA) was the primary endpoint. Because Kaplan–Meier curves crossed, a landmark, time‐varying analysis was used alongside standard tests.

**Results:**

Six‐month WBL averaged 71.4% (70% group) and 62.2% (62% group). Both groups lost similar correction by 10 years (−5.5 vs. −4.8 percentage points, *p* = 0.429), leaving 45% of the 62% group undercorrected versus 13% of the 70% group (*p* < 0.001). Undercorrected knees were converted to TKA more often than normally aligned knees (36.7% vs. 9.6%, *p* = 0.012). The proportional hazards assumption was violated for the group effect (*p* = 0.0025); after 10 years, conditional on event‐free survival, the hazard ratio for 70% versus 62% was 0.54 (95% confidence interval 0.245–1.182, *p* = 0.123). Overall survivorship was similar (22.0% vs. 24.5% converted, *p* = 0.954), but this comparison was underpowered (4.8%) and should not be read as equivalence. Clinical scores did not differ significantly.

**Conclusions:**

Both targets lost similar correction over 10 years, but the 62% target was closer to the undercorrection range, linked to more TKA conversion. Hazards were not proportional over time: The 70% target carried an early, overcorrection‐related risk, while the 62% target carried a later, undercorrection‐related risk undetectable by an overall Cox comparison alone. A target close to, but not exceeding, 70% may lower the risk of both patterns, pending confirmation with an intermediate target and a larger, adequately powered sample.

**Level of Evidence:**

Level II, retrospective long‐term follow‐up analysis of a prospectively randomized cohort.

## Graphical abstract

This 10‐year randomized comparison of 103 knees undergoing medial opening wedge high tibial osteotomy (MOWHTO) evaluated two intraoperative weight‐bearing line (WBL) correction targets, 70% (*n* = 50) and 62% (*n* = 53). Both targets lost a similar amount of correction by 10 years, but because the 62% target starts closer to the undercorrection threshold, this loss left 45% of that group undercorrected, compared with 13% of the 70% group. Undercorrected knees were converted to total knee arthroplasty (TKA) more often than normally aligned knees (36.7% vs. 9.6%, *p* = 0.012). Kaplan–Meier curves crossed: The 70% group had more early, overcorrection‐related failures, while the 62% group had later, undercorrection‐related failures and formal testing confirmed the two targets did not carry a constant relative risk over time. Overall long‐term survivorship was similar between targets, but this comparison was underpowered and should not be read as evidence of equivalence. These findings suggest that a target near, but not exceeding, 70% may reduce exposure to both failure patterns—a hypothesis that should be confirmed in a prospective study with an intermediate target.

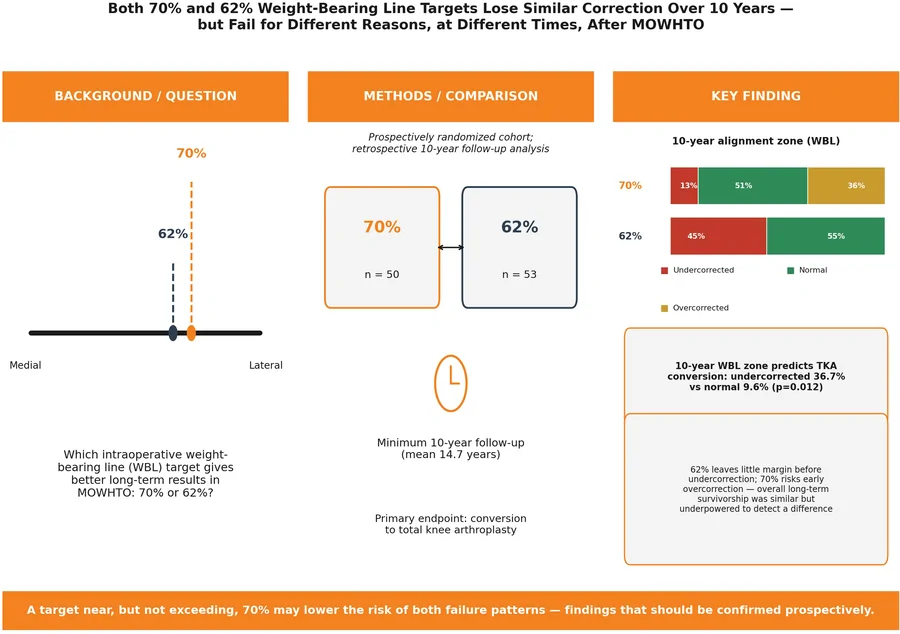

**Abbreviations**
ACL: anterior cruciate ligament
BMI: body mass index
CI: confidence interval
HKA: hip–knee–ankle
KSFS: Knee Society Function Score
KSS: Knee Society Score
MOWHTO: medial opening wedge high tibial osteotomy
MRI: magnetic resonance imaging
OKS: Oxford Knee Score
SD: standard deviation
TKA: total knee arthroplasty
WBL: weight‐bearing line
WOMAC: Western Ontario and Mcmaster Universities Osteoarthritis Index

## INTRODUCTION

Medial opening wedge high tibial osteotomy (MOWHTO) is a well‐established treatment for varus malalignment with medial compartment osteoarthritis. Its long‐term success depends heavily on the alignment achieved at the time of surgery, since both undercorrection and overcorrection can shift load onto a compartment that is not designed to bear it.

Opinions on the ideal correction target have shifted over time. The Fujisawa point, near the 62% weight‐bearing line (WBL), was long considered the standard target [7], but more recent work has favoured staying at or below the traditional Fujisawa point [8]. At the same time, overcorrection has been linked to patellofemoral joint problems [15] and lower patient satisfaction [14]. Most comparative studies of correction targets report outcomes at only 5–7 years, and it is well recognized that alignment continues to change after surgery as the plate, bone and surrounding soft tissue settle.

What remains unclear is whether this postoperative change in alignment follows a similar pattern regardless of the initial target, and how any differences manifest in alignment and implant survivorship beyond the first decade. Without long‐term data, a target chosen to look favourable at 2–5 years could still leave a knee vulnerable to failure later on.

This study aimed to compare 10‐year radiographic and clinical outcomes between two intraoperative WBL correction targets, 70% and 62%, after MOWHTO and to describe how alignment in each group changed between 6 months and 10 years.

It was hypothesized that the 70% target would result in a lower rate of late undercorrection and TKA conversion than the 62% target, without a higher rate of overall long‐term failure.

## METHODS

### Study design

This study was a retrospective long‐term follow‐up analysis of a prospectively randomized cohort; the original treatment allocation was performed prospectively on the day of admission using a computer‐generated randomization sequence, whereas the long‐term clinical and radiographic outcomes were subsequently retrieved and analysed retrospectively. Patients who underwent MOWHTO between 2009 and 2011 were randomized 1:1 to a target WBL correction of 70% or a conventional target of 62%.

### Randomization and blinding

One hundred forty consecutive patients undergoing primary MOWHTO were randomized in a 1:1 ratio (70 to the 70% WBL target, 70 to the 62% WBL target) using a computer‐generated randomization sequence, generated by a researcher involved in the study, on the day of admission before surgery, according to the predefined institutional protocol. Formal allocation concealment was not implemented; the randomization sequence was accessible to the study team. The surgeon and surgical team were not blinded to the assigned target, since this was required for preoperative planning. Two researchers who performed radiographic measurements were not blinded to group allocation. Clinical outcomes were recorded by a third researcher who did not have access to the assigned correction target and was therefore blinded to treatment allocation; where a patient could not attend in person, this researcher conducted a telephone assessment at the designated follow‐up time.

### Patient selection

**Figure 1:**
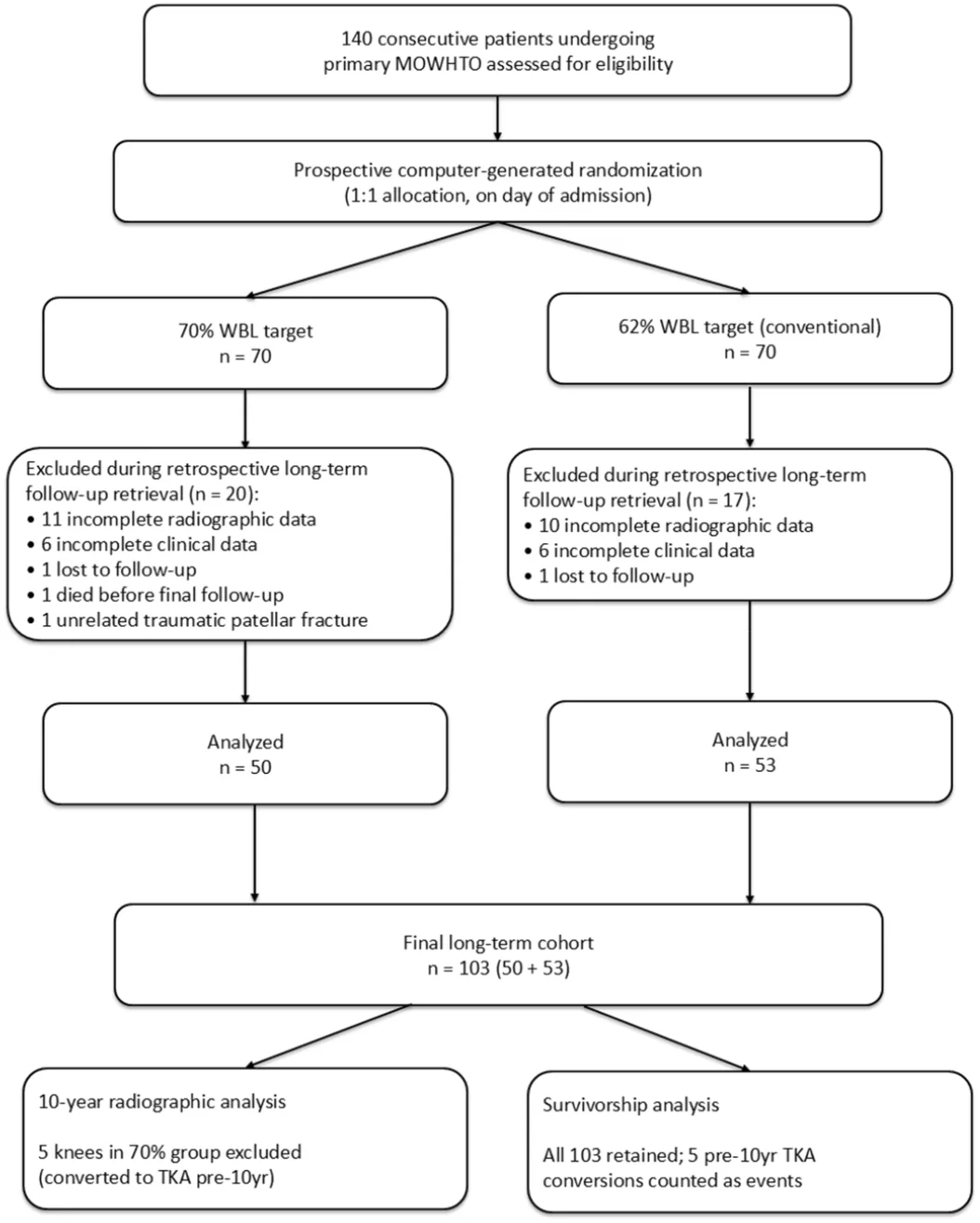
CONSORT‐style flow diagram: original randomization (*n* = 140, 1:1) and exclusions during long‐term follow‐up retrieval, resulting in the final analysed cohort (*n* = 103). MOWHTO, medial opening wedge high tibial osteotomy; TKA, total knee arthroplasty; WBL, weight‐bearing line.

All procedures were performed by a single senior surgeon using the biplanar open‐wedge technique with a locking plate (TomoFix) [16, 19], and all patients followed the same postoperative rehabilitation protocol. Preoperative correction planning used the Miniaci method [18] in both groups, so the only intended difference between groups was the WBL target. Of the 140 originally randomized patients, the final long‐term analysis included 103 knees in 101 patients: 50 knees in the 70% group and 53 knees in the 62% group, after exclusions during retrospective retrieval of long‐term data (detailed in Results, Patient flow section; Figure 1). Four patients contributed two knees each through staged bilateral MOWHTO; two of these patients had one knee assigned to each target, and two had both knees assigned to the same target.

### Inclusion criteria

1. Primary MOWHTO for medial compartment osteoarthritis.
2. Minimum 10‐year follow‐up.
3. Complete clinical and radiographic data.

### Exclusion criteria

1. History of ipsilateral limb surgery or fracture.
2. Inflammatory or post‐traumatic arthritis.
3. Lost to follow‐up or death.
4. Unrelated trauma after index surgery and affecting outcome assessment.
5. Lateral compartmental, patellofemoral or tricompartmental osteoarthritis.

### Surgical technique

MOWHTO was performed with the biplanar open‐wedge technique and a TomoFix locking plate [16, 19]. The Miniaci method [18] was used for preoperative correction planning in both groups. Intraoperative correction was guided by fluoroscopy toward the assigned WBL target, 70% or 62% [10, 13].

### Rehabilitation protocol

All patients followed the same standardized rehabilitation protocol. Range‐of‐motion exercises began on the first postoperative day. Protected weight bearing was permitted after 6 weeks, once radiographs showed signs of union. Supervised physiotherapy continued for 3 months after surgery.

### Radiographic evaluation

**Figure 2:**
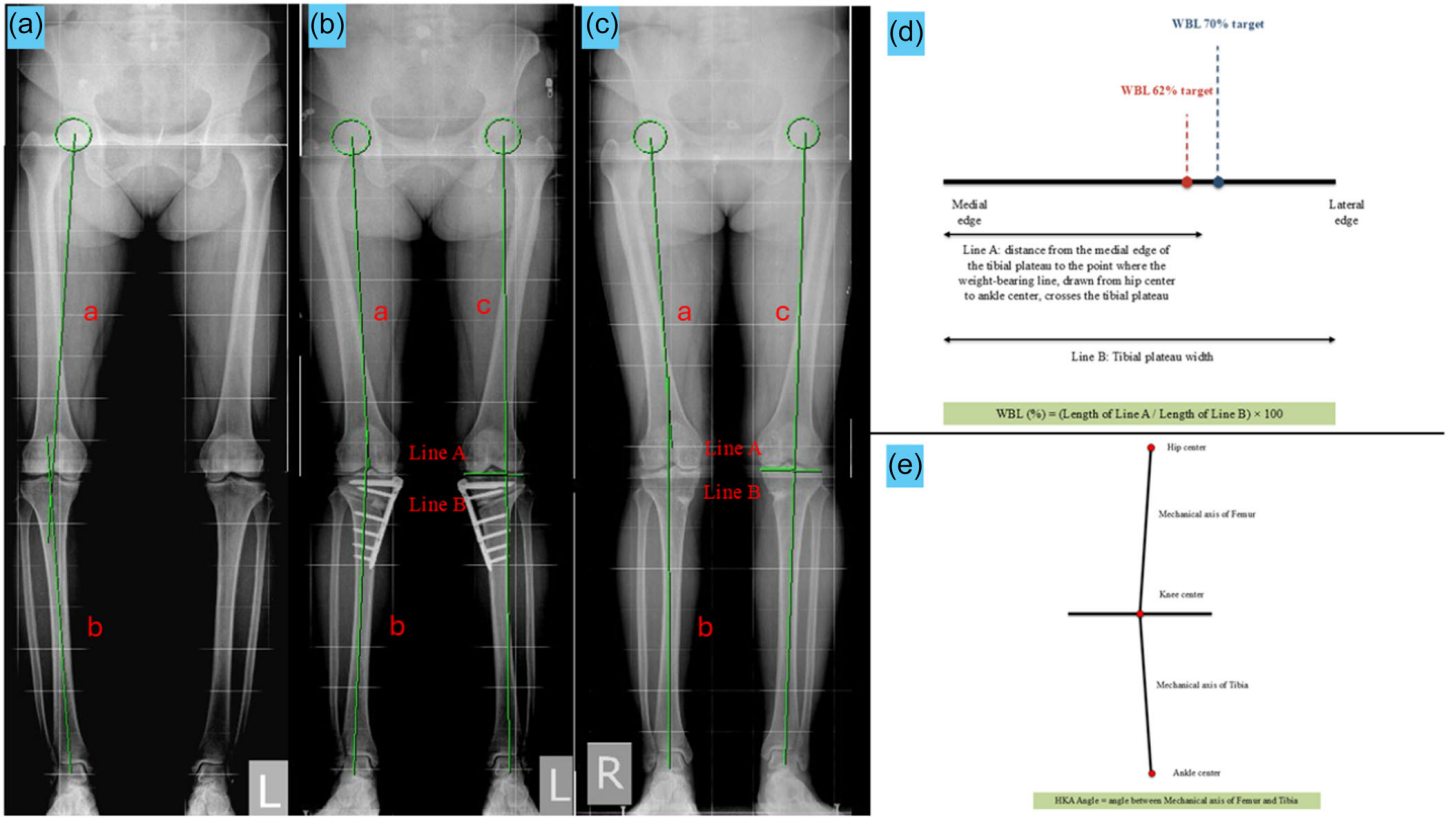
Radiographic (a–c) and schematic (d, e) demonstration of the two primary radiographic measurements: hip–knee–ankle (HKA) angle measured on right knee at before surgery (a), 6 months after surgery (b), 10 years after surgery (c); weight‐bearing line (WBL) measured on left knee at 6 months after surgery (b), 10 years after surgery (c), with the 62% and 70% target points shown (d).

Hip–knee–ankle (HKA) angle was measured before surgery, at 6 months, and at 10 years; WBL was measured at 6 months and at 10 years, using a standard weight‐bearing long film scannogram (Figure 2). WBL was expressed as a percentage of tibial plateau width (Figure 2). Loss of correction was defined as a change in WBL toward varus between 6 months and 10 years, and overcorrection as a change toward valgus over the same interval. At ten years, using the target range defined by the Miniaci method [17] and described by Lobenhoffer et al. [16], patients were categorized into undercorrected (WBL < 60%), normal (WBL 60%–70%) and overcorrected (WBL > 70%), and separately, by HKA angle using criteria from a related MOWHTO alignment study [11] (undercorrected <180°, normal 180°–186°, overcorrected >186°). Interobserver reliability of the radiographic measurements was assessed using a two‐way random‐effects, single‐measure, absolute‐agreement intraclass correlation coefficient (ICC [1, 2]) with 95% confidence intervals (CIs), calculated separately for each group.

### Clinical evaluation

Knee Society Score (KSS) and Knee Society Function Score (KSFS) were recorded before surgery and at 10 years. Oxford Knee Score (OKS) [6] and Western Ontario and McMaster Universities Osteoarthritis Index (WOMAC) were recorded at 10 years. Lateral compartment status was assessed using a combined magnetic resonance imaging (MRI) and radiographic evaluation, performed preoperatively and at 10‐year follow‐up in all 103 patients. MRI was performed on a 1.5‐T system, with sagittal, coronal and axial proton‐density sequences, T1‐weighted, T2‐weighted and fat‐suppressed sequences. Patients with preoperative lateral‐compartment or patellofemoral osteoarthritis were excluded (see Exclusion criteria section); MRI at 10 years was therefore used, together with standing long‐leg, anteroposterior standing and varus/valgus stress radiographs, to classify the lateral compartment qualitatively as good/preserved or arthritic, rather than to generate a formal cartilage grading score (e.g., Outerbridge, International Cartilage Repair Society [ICRS], Whole‐Organ Magnetic Resonance Imaging Score [WORMS] or MRI Osteoarthritis Knee Score [MOAKS]). Two researchers assessed the imaging independently and were blinded to correction‐target allocation; agreement between readers was 100%, and no formal interobserver statistic (e.g., kappa) was calculated. Meniscal status was assessed preoperatively by MRI and confirmed at surgery by diagnostic arthroscopy, and any meniscal procedure performed was recorded.

### Outcome measures

The primary outcome was conversion to TKA. Secondary outcomes were 10‐year KSS, KSFS, OKS and WOMAC scores; 10‐year WBL and HKA alignment zone; and cause of TKA conversion.

### Follow‐up

All patients were followed for a minimum of 10 years. Total observation time to TKA conversion or last follow‐up averaged 14.7 years (3.9–17.4 years).

### Failure definition

The primary failure endpoint was conversion to TKA, recorded with the date of conversion. The cause of failure for each converted knee was determined independently by two reviewers blinded to group allocation, based on operative and imaging records; the two reviewers agreed on every case. Five knees, all in the 70% group, were converted to TKA before their scheduled 10‐year radiographic assessment; these knees were not excluded from the study cohort, but had no native‐knee value available for the 10‐year WBL/HKA comparison and were therefore excluded from that specific comparison only, while being retained in the survivorship analysis as an event. This is a potential source of selection bias in the 10‐year radiographic comparison, discussed further in Limitations.

### Statistical analysis

Continuous variables were tested for normality with the Shapiro–Wilk test and were compared between groups with the independent‐samples *t* test for normal distribution or the Mann–Whitney *U* test for non‐normal distribution. Categorical variables were compared with the *χ* ^2^ test or Fisher's exact test where expected cell counts were below 5. Within‐group change in WBL from 6 months to 10 years was assessed with the Wilcoxon signed‐rank test. The association between 10‐year alignment zone and TKA conversion was assessed with the *χ* ^2^ test. TKA‐free survivorship was estimated with the Kaplan–Meier method and compared between groups with the log‐rank test. Because the Kaplan–Meier curves crossed, the proportional hazards assumption for the group covariate was tested using Schoenfeld residuals (correlation with time). Given the limited number of events, a likelihood ratio (LR) test was additionally used to compare a standard constant‐hazard ratio (HR) Cox model against a time‐varying‐coefficient model in which the group effect was allowed to differ before and after the 10‐year time point at which the curves crossed. Where the proportional hazards assumption was violated, group comparisons were presented as landmark analyses (log‐rank test up to 10 years; HR conditional on event‐free survival to 10 years, thereafter) rather than as a single overall HR. A multivariable Cox proportional hazards model was additionally used, adjusting for age [1], body mass index (BMI), preoperative Kellgren–Lawrence grade [12] and preoperative HKA angle, acknowledging the same proportional hazards limitation. A two‐sided *p* value below 0.05 was considered statistically significant.

### Sample size and power

Sample size was fixed from existing retrospective cohorts (*n* = 50 and *n* = 53) rather than calculated in advance. Post hoc power was estimated using a two‐proportion *z* test approximation (two‐sided *α* = 0.05). The study had 94.8% power in detecting the observed difference in 10‐year WBL undercorrection rate (13% vs. 45%), but only 4.8% power in detecting the observed difference in overall TKA conversion rate (22.0% vs. 24.5%). With the achieved sample sizes, the study had 80% power to detect a proportion difference of about 26 percentage points or a standardized effect size (Cohen's *d*) of about 0.55 or larger.

## RESULTS

### Patient flow

During the study period, 140 consecutive patients undergoing primary MOWHTO were randomized 1:1 to either the 70% WBL target group (*n* = 70) or the conventional (62%) correction target group (*n* = 70), using a computer‐generated randomization sequence (Figure 1). During retrospective retrieval of long‐term outcomes, 20 patients were excluded from the 70% group (11 for incomplete radiographic data, 6 for incomplete clinical data, 1 lost to follow‐up, 1 who died before final follow‐up and 1 with a traumatic patellar fracture unrelated to the index procedure), leaving 50 patients analysed. In the 62% group, 17 patients were excluded (10 for incomplete radiographic data, 6 for incomplete clinical data, 1 lost to follow‐up), leaving 53 patients analysed. The final long‐term cohort comprised 103 patients: 50 in the 70% group and 53 in the 62% group. All 103 patients had a minimum of 10 years of follow‐up; total observation time averaged 14.7 years (median 15.1, range: 3.9–17.4 years). Five knees, all in the 70% group, were converted to TKA before their scheduled 10‐year radiographic assessment; these were not excluded from the cohort, but had no native‐knee value available and were therefore excluded from the 10‐year WBL and HKA comparisons only, while being retained in the survivorship analysis as an event.

### Radiographic measurement methodology and reliability

Interobserver reliability for HKA angle and WBL measurements was excellent in both groups. ICC(2,1) point estimates ranged from 0.982 to 0.996 across preoperative, 6‐month and 10‐year measurements, with 95% CIs ranging from 0.972 to 0.997 (full group‐specific values provided in Table S1).

### Baseline characteristics

Baseline demographic, radiological and clinical data were similar between groups (Table 1). No significant differences were found in age, height, weight, BMI, preoperative HKA angle, preoperative KSS, preoperative KSFS, sex distribution or preoperative Kellgren–Lawrence grade.

**Table 1:** Baseline demographic, radiological and clinical comparability between groups.

Variable	70% group (*n* = 50)	62% group (*n* = 53)	Test	*p* value
Age, years	57.4 ± 7.8	58.3 ± 7.2	*t* test	0.554
Height, cm	158.0 ± 7.3	158.2 ± 6.5	Mann–Whitney *U*	0.587
Weight, kg	63.2 ± 10.0	64.3 ± 9.6	Mann–Whitney *U*	0.685
BMI, kg/m^2^	25.4 ± 3.6	25.5 ± 3.3	*t* test	0.829
Preoperative HKA, °	175.4 ± 3.0	175.2 ± 2.7	Mann‐Whitney *U*	0.466
Preoperative KSS	66.8 ± 9.5	66.3 ± 9.5	Mann–Whitney *U*	0.897
Preoperative KSFS	52.3 ± 11.5	52.3 ± 13.1	Mann–Whitney *U*	0.843
Sex, F/M	43/7	45/8	Fisher exact	1.000
Kellgren–Lawrence grade, 2/3	33/17	36/17	Fisher exact	1.000

*Note*: Values are mean ± SD unless otherwise stated.

Abbreviations: BMI, body mass index; F/M, female/male; HKA, hip–knee–ankle; KSFS, Knee Society Function Score; KSS, Knee Society Score; SD, standard deviation.

### Clinical outcomes

Ten‐year KSS, KSFS, OKS and WOMAC scores did not differ significantly between groups, nor did the change in KSS or KSFS from before surgery to 10 years (Table 2).

**Table 2:** Ten‐year clinical outcome scores by group.

Score	70% group	62% group	Test	*p* value
10‐year KSS	89.4 ± 12.8	91.0 ± 9.3	Mann–Whitney *U*	0.906
10‐year KSFS	73.5 ± 17.8	75.7 ± 16.5	Mann–Whitney *U*	0.997
10‐year OKS	41.2 ± 4.0	40.5 ± 4.1	Mann–Whitney *U*	0.281
10‐year WOMAC	4.1 ± 7.9	4.3 ± 4.7	Mann–Whitney *U*	0.147
Change in KSS (10 years − preoperative)	22.5 ± 15.9	24.7 ± 13.1	*t* test	0.456
Change in KSFS (10 years − preoperative)	21.2 ± 22.0	23.4 ± 22.5	Mann–Whitney *U*	0.873

*Note*: Values are mean ± SD; *n *= 50 (70% group), *n* = 53 (62% group). Ten‐year KSS, WOMAC and OKS also did not differ significantly by 10‐year alignment zone (Kruskal–Wallis *p* = 0.717, 0.343 and 0.920, respectively).

Abbreviations: KSFS, Knee Society Function Score; KSS, Knee Society Score; OKS, Oxford Knee Score; SD, standard deviation; WOMAC, Western Ontario and McMaster Universities Osteoarthritis Index.

### Radiographic outcomes

Radiographic measurements at each time point are summarized in Table 3. Preoperative HKA angle and WBL were similar between groups. At 6 months, there were significantly different HKA angles and WBL between groups, confirming that the assigned intraoperative targets were achieved (WBL 71.4% ± 3.2% vs. 62.2% ± 2.1%, *p* < 0.001). By 10 years, WBL and HKA angles had decreased from their 6‐month values in both groups, reflecting loss of correction. The size of this loss from 6 months to 10 years was similar between groups for both WBL (−5.5 vs. −4.8 percentage points, *p* = 0.429) and HKA angle (−1.6° vs. −2.0°, *p* = 0.328).

**Table 3:** Radiographic outcomes (HKA angle and WBL) at preoperative, 6‐month and 10‐year time points, and loss of correction (change from 6 months to 10 years).

Measurement	70% group	62% group	Test	*p* value
Preop HKA, °	175.4 ± 3.0 (*n* = 50)	175.2 ± 2.7 (*n* = 53)	Mann–Whitney *U*	0.466
6‐month HKA, °	184.9 ± 1.2 (*n* = 50)	183.2 ± 1.5 (*n* = 53)	*t* test	<0.001
10‐year HKA, °	183.3 ± 2.4 (*n* = 45)	181.2 ± 2.9 (*n* = 53)	Mann–Whitney *U*	<0.001
6‐month WBL, %	71.4 ± 3.2 (*n* = 50)	62.2 ± 2.1 (*n* = 53)	Mann–Whitney *U*	<0.001
10‐year WBL, %	65.6 ± 6.9 (*n* = 45)	57.4 ± 5.9 (*n* = 53)	Mann–Whitney *U*	<0.001
Change in HKA, 6 months to 10 years, °	−1.6 ± 2.0 (*n* = 45)	−2.0 ± 2.4 (*n* = 53)	*t* test	0.328
Change in WBL, 6 months to 10 years, %	−5.5 ± 6.1 (*n* = 45)	−4.8 ± 5.6 (*n* = 53)	Mann–Whitney *U*	0.429

*Note*: Values are mean ± SD.

Abbreviations: HKA, hip–knee–ankle; SD, standard deviation; WBL, weight‐bearing line.

Because the two groups started from different baselines, this similar loss of correction led to markedly different 10‐year alignment zone distributions (Table 4). In the 70% group, 51% of knees remained in the normal zone, and 36% moved into mild overcorrection, with 13% undercorrected. In the 62% group, 55% remained in the normal zone, none were overcorrected, and 45% moved into undercorrection (*p* < 0.001).

**Table 4:** Ten‐year WBL alignment zone by group (*χ* ^2^ *p* < 0.001).

WBL zone at 10 years	70% group, *n* (%)	62% group, *n* (%)
Undercorrected (WBL < 60%)	6 (13%)	24 (45%)
Normal (WBL 60%–70%)	23 (51%)	29 (55%)
Overcorrected (WBL > 70%)	16 (36%)	0 (0%)
Total evaluable	45	53

*Note*: Knees converted to TKA before their 10‐year radiograph (*n* = 5, all in the 70% group) are excluded, as no native‐knee 10‐year value exists—see Limitations for discussion of the resulting selection bias.

Abbreviations: TKA, total knee arthroplasty; WBL, weight‐bearing line.

Ten‐year WBL alignment zone shows significant association with subsequent TKA conversion (Table 5): 36.7% of undercorrected knees (11/30) were later converted to TKA, compared with 9.6% of normally aligned knees (5/52) and 18.8% of overcorrected knees (3/16) (*p* = 0.012).

**Table 5:** Subsequent TKA conversion by 10‐year WBL‐based alignment zone, among knees followed to 10‐year assessment (*p* = 0.012).

10‐year WBL zone	No subsequent TKA	Subsequent TKA	Conversion rate
Undercorrected	19	11	36.7%
Normal	47	5	9.6%
Overcorrected	13	3	18.8%

Abbreviations: TKA, total knee arthroplasty; WBL, weight‐bearing line.

Ten‐year alignment was also classified with HKA angle, using criteria applied in a related MOWHTO alignment study [11]: undercorrected HKA < 180°, normal HKA 180°–186° and overcorrected HKA > 186°. This classification also differed significantly by group (*p* = 0.023): The 62% group clustered in the undercorrected/normal range (52/53, 98%, with only one overcorrected knee), while the 70% group showed a wider spread, including more overcorrected knees (5/45, 11%) (Table 6).

**Table 6:** Ten‐year HKA angle‐based alignment zone by group (*p* = 0.023).

HKA zone at 10 years	70% group, *n* (%)	62% group, *n* (%)
Undercorrected (HKA < 180°)	3 (7%)	12 (23%)
Normal (HKA 180–186°)	37 (82%)	40 (75%)
Overcorrected (HKA > 186°)	5 (11%)	1 (2%)
Total evaluable	45	53

Abbreviation: HKA, hip–knee–ankle.

The HKA‐based classification showed the same association with subsequent TKA conversion (*p* = 0.047), matching the WBL‐based finding: 40.0% of undercorrected knees (6/15) were later converted to TKA, compared with 14.3% of normal‐zone knees (11/77) and 33.3% of overcorrected knees (2/6) (Table 7).

**Table 7:** Subsequent TKA conversion by 10‐year HKA‐based alignment zone (*p* = 0.047).

10‐year HKA zone	No subsequent TKA	Subsequent TKA	Conversion rate
Undercorrected	9	6	40.0%
Normal	66	11	14.3%
Overcorrected	4	2	33.3%

Abbreviations: HKA, hip–knee–ankle; TKA, total knee arthroplasty.

### Complications

**Figure 3:**
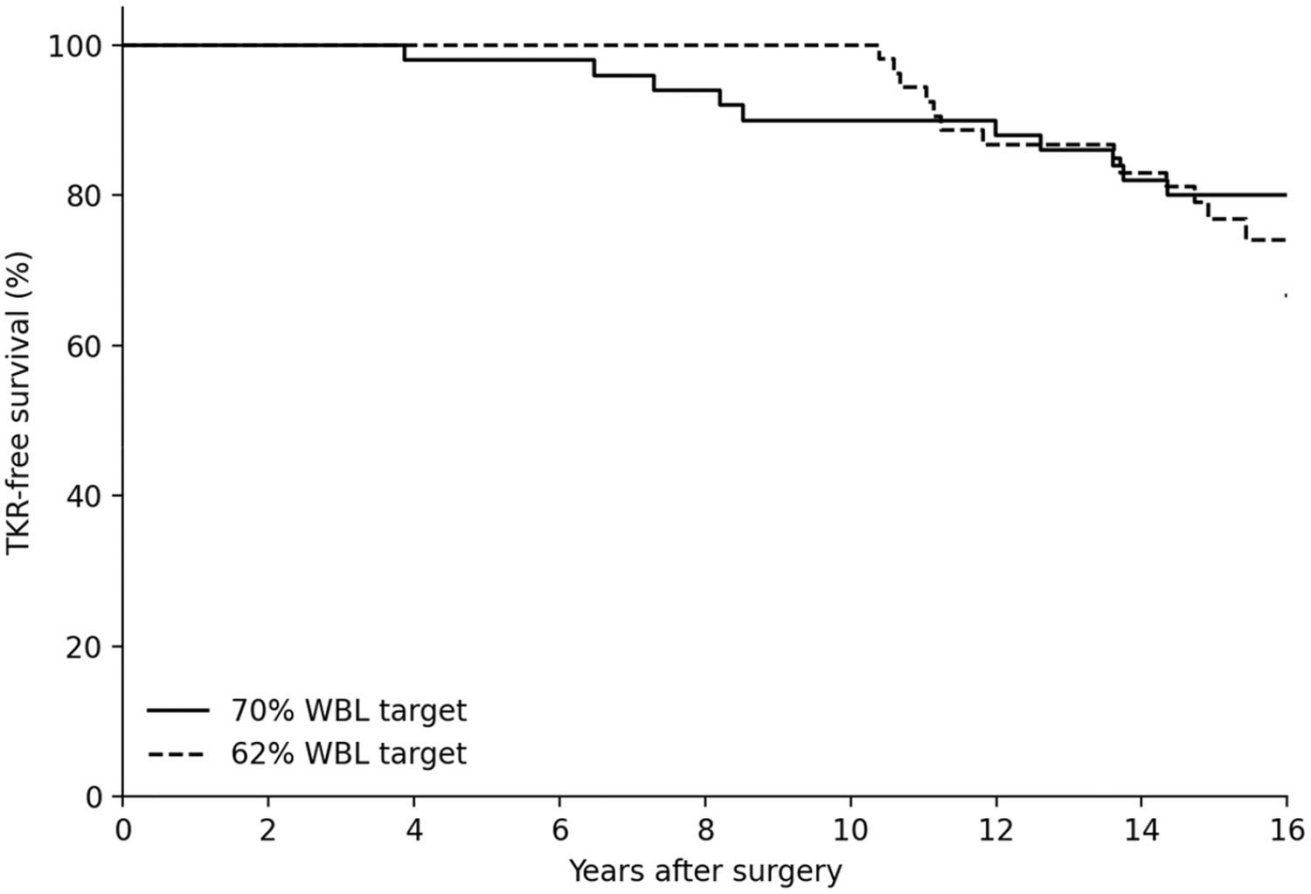
Kaplan–Meier TKA‐free survival curves by group, full follow‐up. Curves cross near the 10‐year time point, consistent with the non‐proportional hazards identified statistically (see Complications and Discussion sections). TKA, total knee arthroplasty; WBL, weight‐bearing line.

Overall, 24 out of 103 knees (23.3%) were converted to TKA during follow‐up: 11 out of 50 (22.0%) in the 70% group and 13 out of 53 (24.5%) in the 62% group. Because the Kaplan–Meier curves crossed (Figure 3), the proportional hazards assumption was tested and found to be violated for the group effect (LR test comparing a constant‐HR model against a time‐varying model split at the 10‐year landmark: LR = 9.11, df = 1, *p* = 0.0025; Schoenfeld residual correlation with time, *p* = 0.177, likely underpowered given only 24 events). A single overall Cox HR for the group is therefore not an appropriate summary of this comparison and is reported below for completeness alongside the more appropriate landmark analysis. Before 10 years, TKA‐free survivorship differed significantly between groups (90.0% in the 70% group vs. 100.0% in the 62% group, landmark log‐rank *p* = 0.019); this comparison could not be summarized as an HR because all five conversions before 10 years occurred in the 70% group. After 10 years, conditional on event‐free survival to that point, the HR for the 70% group versus the 62% group was 0.54 (95% CI 0.245–1.182, *p* = 0.123). Over the full follow‐up period, the survival curves converged and crossed, and the overall log‐rank test was not significant (*p* = 0.954; Table 8, Figure 3). Because post hoc power for this overall comparison was only 4.8%, this finding should not be interpreted as evidence that the two targets produce equivalent long‐term failure risk.

**Table 8:** Kaplan–Meier TKA‐free survivorship at fixed time points, full follow‐up.

Follow‐up	70% group TKA‐free survival	62% group TKA‐free survival
5 years	98.0%	100.0%
10 years	90.0%	100.0%
12 years	88.0%	86.8%
15 years	80.0%	76.9%

Abbreviation: TKA, total knee arthroplasty.

The pattern of failure differed between groups. All five early (<10‐year) conversions in the 70% group were attributed to causes consistent with overcorrection: patellofemoral joint osteoarthritis, partial or complete anterior cruciate ligament (ACL) tear with patellofemoral joint osteoarthritis, explicit overcorrection and general osteoarthritis progression. No conversions occurred before 10 years in the 62% group; of the 13 eventual conversions in this group, 12 were attributed to loss of correction with progression of medial osteoarthritis, consistent with a late, undercorrection‐driven failure pattern.

### Additional analyses

In multivariable Cox proportional hazards regression analyses for TKA conversion over the full follow‐up period, adjusting for age, BMI, preoperative Kellgren–Lawrence grade and preoperative HKA angle, correction target group (70% vs. 62%) was not an independent predictor of TKA conversion (HR 1.03, 95% CI 0.46–2.32, *p* = 0.945). None of the adjustment covariates reached statistical significance.

A sensitivity analysis restricted to one knee per patient (the earlier‐operated knee, *n* = 99) reproduced both the 10‐year alignment zone distribution (*p* < 0.001) and the zone–TKA conversion association (*p* = 0.012), indicating the main findings were not driven by the paired bilateral knees.

Lateral compartment condition at 10 years, assessed with MRI and stress radiographs, was rated as good in 47/50 knees in the 70% group and 50/53 knees in the 62% group, with no significant difference between groups (three knees with osteoarthritis in each group; *p* = 1.000). Preoperative meniscal pathology was almost similar in both groups (only one knee overall had an intact meniscus), which limited meaningful comparison. The type of meniscal procedure performed did not show a statistically significant difference between groups (*p* = 0.211).

### Discussion

Both intraoperative WBL correction targets, 70% and 62%, lost a similar amount of correction over 10 years, around 5 percentage points. Because the 62% target sits close to the threshold for undercorrection, this loss pushed nearly half of these knees into an undercorrected alignment zone by 10 years, more than three times the rate seen in the 70% group. Landing in the undercorrected zone at 10 years was linked to a significantly higher rate of subsequent TKA conversion. Overall long‐term survivorship appeared similar between groups, but this comparison was markedly underpowered (4.8% post hoc power) and should not be read as evidence of equivalence. More importantly, the Kaplan–Meier curves crossed, and formal testing confirmed that the proportional hazards assumption did not hold for the group effect (*p* = 0.0025); the two groups therefore did not fail at a constant relative rate over time, but instead followed two different failure patterns on different timelines: early, overcorrection‐related failure in the 70% group, and later, undercorrection‐related failure in the 62% group. Clinical outcome scores at 10 years did not differ significantly between groups.

These findings agree with a related long‐term MOWHTO series [11]; that study likewise found that non‐optimally aligned knees lost more correction and converted to TKA significantly earlier, despite similar patient‐reported outcomes among knees that kept their native joint. In the present study, overall long‐term survivorship reports were 76%–80% TKA‐free at 15 years, which falls within the range reported by other long‐term MOWHTO series (79%–97.6% TKA‐free at 10 years) [2, 3, 4]. The early, overcorrection‐related failure pattern in the 70% group fits reports linking overcorrection to worse patient‐reported outcomes [14], while the undercorrection‐related pattern in the 62% group shows the classic association between residual undercorrection and failure described by Coventry [5]. Recent literature continues to reflect this tension between correction strategies, including work on individualized, lower‐target alignment [8, 9] and on functional and gait‐based outcomes after HTO [20], underscoring that no single fixed target has been established as universally optimal.

These results carry a practical message for choosing a correction target. Aiming for 62% leaves little margin before the loss of correction observed in this cohort pushes the knee into the undercorrected zone, which had the highest TKA conversion rate on both the WBL‐ and HKA‐based classifications (Tables 7 and 8). Aiming for 70% avoided this pattern without increasing overall long‐term failure, since full‐follow‐up survivorship was similar between groups (Table 6). This protection, however, was specific to late (≥10‐year) undercorrection‐related failure: The 70% group instead showed an early, overcorrection‐related failure pattern not seen with 62%, so protection against late failure should not be considered as lower failure overall, and the overall equivalence in survivorship reflects an underpowered comparison of two different failure timelines rather than genuinely equal risk. Because the undercorrected zone had a higher conversion rate than the overcorrected zone on both classifications, a target close to, but not exceeding, 70% and clearly above 62% may reduce exposure to both failure patterns. Intraoperative error in achieving the desired target, which may fall on either side of the intended value, should also be considered. This inference follows from the two targets tested here; an intermediate target was not directly evaluated, and this specific recommendation should be treated as hypothesis‐generating pending a prospective, adequately powered study.

The similar magnitude of correction loss in both groups suggests that alignment change after MOWHTO reflects a mechanical or biological process, such as plate and bone remodelling or soft tissue equilibration, that proceeds at a broadly similar rate regardless of the initial target. Under this explanation, the choice of intraoperative target mainly sets the safety margin against this expected change rather than altering the change itself. No evidence was found that the 70% target increased lateral compartment degeneration at 10 years on MRI or stress radiographs. This may reflect the modest degree of overcorrection seen in this cohort (mean 10‐year WBL 65.6% in the 70% group); more pronounced overcorrection has been linked to worse outcomes elsewhere [14].

### Strengths

This study has several strengths. The correction target was randomized prospectively at the time of surgery rather than left to the surgeon's discretion, which reduces selection bias compared with a fully retrospective design, although the absence of formal allocation concealment and the lack of blinding for the surgical team and radiographic assessors are acknowledged (see Randomization and blinding section). All procedures were performed by a single surgeon with a standardized technique and rehabilitation protocol. Minimum follow‐up was 10 years, with an average of 14.7 years. The cause of TKA conversion was determined by two reviewers blinded to group allocation, who agreed on every case. The main radiographic finding was confirmed with two independent alignment measures, WBL and HKA angle, using criteria from a related published study [11], and interobserver reliability for these measurements was excellent (ICC 0.982–0.996). A sensitivity analysis confirmed that the main findings were not driven by the small number of patients who contributed bilateral knees.

### Limitations

This study also has several important limitations. It is a retrospective long‐term follow‐up analysis of a single‐surgeon, single‐institution cohort; although the original allocation was randomized, allocation concealment was not formally implemented, and the surgical team and radiographic assessors were not blinded, which may have introduced bias despite randomization. Knees converted to TKA before their 10‐year radiograph could not contribute a native‐knee 10‐year alignment value; because all five such knees were in the 70% group, this is a specific source of selection bias that likely understates the true magnitude of overcorrection and/or correction loss in that group, since these knees plausibly represent the most overcorrected or most rapidly failing cases. The study was well powered (>90%) to detect the observed alignment zone differences but markedly underpowered (4.8%) to detect the observed difference in overall TKA conversion rate; the similar overall survivorship reported here must not be interpreted as evidence of equivalence between targets. In addition, the Kaplan–Meier curves for the two groups crossed, and formal testing confirmed that the proportional hazards assumption was violated for the group effect; the single overall Cox HR and log‐rank test reported for completeness should therefore be interpreted with particular caution, and the landmark‐based analyses are considered more informative for this comparison. Meniscal and lateral‐compartment comparisons were limited by near‐ceiling preoperative meniscal pathology and low event counts, respectively, and lateral‐compartment assessment relied on a qualitative, non‐formally‐graded classification with 100% but non‐statistically‐quantified reader agreement.

### Future directions

A prospective study using an intermediate correction target, between 62% and 70%, is needed to test the target‐selection recommendation raised by this study directly. Multi‐centre validation would help address the single‐surgeon, single‐institution design. Once validated minimal clinically important difference and substantial clinical benefit thresholds for KSS, OKS and WOMAC in the MOWHTO population become available, applying them to this cohort's clinical scores would add further context to the finding that scores did not differ significantly between groups. Based on the findings of this study, the treating institution has since adopted a target range of 65%–70% WBL rather than a single fixed correction point, with plans to evaluate this range‐based approach prospectively in a future study.

## CONCLUSIONS

In this 10‐year comparative study, MOWHTO patients targeted to 70% WBL and 62% WBL lost a similar amount of correction over time, but the 62% target's closeness to the undercorrection threshold led more knees to drift into undercorrection by 10 years, which was linked to more subsequent TKA conversion. The 70% target instead carried an early, overcorrection‐related failure risk not seen with the 62% target, without an increase in 10‐year lateral compartment degeneration. Overall long‐term survivorship and 10‐year clinical scores were similar between targets. These findings support building a correction buffer into the intraoperative target to account for the expected long‐term loss of correction, while showing that different targets carry different failure patterns and timelines that only appear with follow‐up beyond 10 years.

## AUTHOR CONTRIBUTIONS

**Woon Hwa Jung**: Conceptualization; resources (lead operating surgeon); supervision; writing—review and editing. **Manan Paneliya**: Conceptualization; methodology; formal analysis; investigation; data curation; writing—original draft; writing—review and editing. **Saksham Goyal**: Validation; writing—review and editing. **Dong Hyun Kim**: Investigation; data curation; conceptualization. **Ryohei Takeuchi**: Methodology; writing—review and editing.

## FUNDING INFORMATION

The authors have no funding to report.

## CONFLICT OF INTEREST STATEMENT

The authors declare no conflicts of interest.

## ETHICS STATEMENT

MURUP IEC, Murup Hospital, Changwon, Republic of Korea. Approval Number: MURUP/IEC/2026/013. Informed consent was obtained from all patients included in this study.

## Supporting information

Supporting File 1.

## Data Availability

Data are available from the corresponding author on reasonable request.
