# Supplementary material for: A long‐term comparison of radiographic and clinical outcomes between 62% and 70% weight‐bearing line targets in medial opening wedge high tibial osteotomy
Source: J Exp Orthop. 2026 Aug 31;13(3):e70900. doi: 10.1002/jeo2.70900 (PMC13528234; doi:10.1002/jeo2.70900)
Supplement: Supplementary file 1 [file JEO2-13-e70900-s001.docx]

# **Supporting Information**

*Manuscript: “A Long-Term Comparison of Radiographic and Clinical Outcomes Between 62% and 70% Weight-Bearing Line Targets in Medial Opening Wedge High Tibial Osteotomy”*

Interobserver reliability was assessed using a two-way random-effects, single-measure, absolute-agreement intraclass correlation coefficient (ICC [2,1]) with 95% confidence intervals, calculated separately for each group and for each radiographic measurement and time point.

| **Group** | **Measurement** | **n** | **ICC (2,1)** | **95% CI** | **Interpretation** |
| --- | --- | --- | --- | --- | --- |
| 62% | HKA, preoperative | 53 | 0.987 | 0.979–0.991 | Excellent |
| 62% | HKA, 6-month | 53 | 0.992 | 0.988–0.994 | Excellent |
| 62% | HKA, 10-year | 53 | 0.989 | 0.978–0.993 | Excellent |
| 62% | WBL, 6-month | 53 | 0.990 | 0.982–0.994 | Excellent |
| 62% | WBL, 10-year | 53 | 0.995 | 0.993–0.996 | Excellent |
| 70% | HKA, preoperative | 50 | 0.989 | 0.981–0.993 | Excellent |
| 70% | HKA, 6-month | 50 | 0.988 | 0.980–0.992 | Excellent |
| 70% | HKA, 10-year | 45 | 0.982 | 0.972–0.988 | Excellent |
| 70% | WBL, 6-month | 50 | 0.995 | 0.992–0.997 | Excellent |
| 70% | WBL, 10-year | 45 | 0.996 | 0.993–0.997 | Excellent |

**Table S1. Interobserver reliability (ICC [2,1]) for hip-knee-ankle (HKA) angle and weight-bearing line (WBL) measurements, by group, measurement, and time point.**
